# An analysis of current practices in undertaking literature reviews in nursing: findings from a focused mapping review and synthesis

**DOI:** 10.1186/s12874-019-0751-7

**Published:** 2019-05-16

**Authors:** Helen Aveyard, Caroline Bradbury-Jones

**Affiliations:** 1 Faculty of Health and Life Sciences, Oxford Brookes University, Jack Straw’s Lane, Oxford, OX3 0FL England, UK; 2 University of Birmingham, Birmingham, England, UK

**Keywords:** Evidence synthesis, Literature review, Meta-ethnography, Systematic review

## Abstract

**Background:**

In this paper we discuss the emergence of many different methods for doing a literature review. Referring back to the early days, when there were essentially two types of review; a Cochrane systematic review and a narrative review, we identify how the term systematic review is now widely used to describe a variety of review types and how the number of available methods for doing a literature review has increased dramatically. This led us to undertake a review of current practice of those doing a literature review and the terms used to describe them.

**Method:**

We undertook a focused mapping review and synthesis. Literature reviews; defined as papers with the terms review or synthesis in the title, published in five nursing journals between January 2017–June 2018 were identified. We recorded the type of review and how these were undertaken.

**Results:**

We identified more than 35 terms used to describe a literature review. Some terms reflected established methods for doing a review whilst others could not be traced to established methods and/or the description of method in the paper was limited. We also found inconsistency in how the terms were used.

**Conclusion:**

We have identified a proliferation of terms used to describe doing a literature review; although it is not clear how many distinct methods are being used. Our review indicates a move from an era when the term narrative review was used to describe all ‘non Cochrane’ reviews; to a time of expansion when alternative systematic approaches were developed to enhance rigour of such narrative reviews; to the current situation in which these approaches have proliferated to the extent so that the academic discipline of doing a literature review has become muddled and confusing. We argue that an ‘era of consolidation’ is needed in which those undertaking reviews are explicit about the method used and ensure that their processes can be traced back to a well described, original primary source.

## Background

Over the past twenty years in nursing, literature reviews have become an increasingly popular form of synthesising evidence and information relevant to the profession. Along with this there has been a proliferation of publications regarding the processes and practicalities of reviewing [1–4], This increase in activity and enthusiasm for undertaking literature reviews is paralleled by the foundation of the Cochrane Collaboration in 1993. Developed in response to the need for up-to-date reviews of evidence of the effectiveness of health care interventions, the Cochrane Collaboration introduced a rigorous method of searching, appraisal and analysis in the form of a ‘handbook’ for doing a systematic review [5] .Subsequently, similar procedural guidance has been produced, for example by the Centre for Reviews and Dissemination (CRD) [6] and The Joanna Briggs Institute [7]. Further guidance has been published to assist researchers with clarity in the reporting of published reviews [8].

In the early days of the literature review era, the methodological toolkit for those undertaking a literature was polarised, in a way that mirrored the paradigm wars of the time within mixed-methods research [9]. We refer to this as the ‘dichotomy era’ (i.e. the 1990s), The prominent methods of literature reviewing fell into one of two camps: The highly rigorous and systematic, mostly quantitative ‘Cochrane style’ review on one hand and a ‘narrative style’ review on the other hand, whereby a body of literature was summarised qualitatively, but the methods were often not articulated. Narrative reviews were particularly popular in dissertations and other student work (and they continue to be so in many cases) but have been criticised for a lack of systematic approach and consequently significant potential for bias in the findings [10, 11].

The latter 1990s and early 2000, saw the emergence of other forms of review, developed as a response to the Cochrane/Narrative dichotomy. These alternative approaches to the Cochrane review provided researchers with reference points for performing reviews that drew on different study types, not just randomised controlled trials. They promoted a systematic and robust approach for all reviews, not just those concerned with effectiveness of interventions and treatments. One of the first published description of methods was Noblet and Hare’s (1998) ‘Meta-ethnography’ [12]. This method, although its name suggests otherwise, could incorporate and synthesise all types of qualitative research, not just ethnographies. The potential confusion regarding the inclusion of studies that were not ethnographies within a meta-ethnography, promoted the description of other similar methods, for example, the meta-synthesis of Walsh and Downe (2005) [13] and the thematic synthesis of Thomas and Harden (2008) [14]. Also, to overcome the dichotomy of the quantitative/qualitative reviews, the integrative review was described according to Whitemore and Knafl (2005) [15]. These reviews can be considered to be literature reviews that have been done in a systematic way but not necessarily adhering to guidelines established by the Cochrane Collaboration. We conceptualise this as the ‘expansion era’. Some of the methods are summarised in Table 1.

**Table 1:** Some examples of different approaches to doing a literature review

Systematic review	This is the ‘original’ systematic review, often responding to ‘does it work’ questions about effectiveness but can be used for a wide range of review questions. The hallmark of a systematic review is that it identifies, appraises and synthesises the empirical evidence that meets pre-specified eligibility criteria. However systematic reviews are not limited to one type of data and can be compiled with quantitative, qualitative research or both. [5] Meta-ethnography, other qualitative synthesis and integrative reviews share many of the characteristics of a systematic review as regards rigour and systematic processes they employ. [12–15]
Scoping review	This is a mapping review, that aims to determine the range of research and other evidence that is available on a topic. A scoping review does not typically include appraisal or analysis [16].
Realist review	This review is a more complex enquiry than a simple ‘does it work?’ question; instead researchers explore why something works, in what circumstances it works and with whom Iit works [17]
Rapid review	These are new review types developed in response to the need to provide a quick evidence base; the methods are largely undefined [18]
Focused mapping review and synthesis (FMRS)	The FMRS is a method of investigating trends in academic publications and is another example of a new type of review. The FMRS was developed in response to the need for a scholarly approach to the identification of trends and is used by those who are exploring methods used in a particular area. [19]

Over the past two decades there has been a proliferation of review types, with corresponding explosion of terms used to describe them. A review of evidence synthesis methodologies by Grant and Booth in 2009 [20] identified 14 different approaches to reviewing the literature and similarly, Booth and colleagues [21] detailed 19 different review types, highlighting the range of review types currently available. We might consider this the ‘proliferation era’. This is however, somewhat a double-edged sword, because although researchers now have far more review methods at their disposal, there is risk of confusion in the field. As Sabatino and colleagues (2014) [22] have argued, review methods are not always consistently applied by researchers.

Aware of such potential inconsistency and also our own confusion at times regarding the range of review methods available, we questioned what was happening within our own discipline of nursing. We undertook a snap-shot, contemporary analysis to explore the range of terms used to describe reviews, the methods currently described in nursing and the underlying trends and patterns in searching, appraisal and analysis adopted by those doing a literature review. The aim was to gain some clarity on what is happening within the field, in order to understand, explain and critique what is happening within the proliferation era.

## Methods

In order to explore current practices in doing a literature review, we undertook a ‘Focused Mapping Review and Synthesis’ (FMRS) – an approach that has been described only recently. This form of review [19] is a method of investigating trends in academic publications and has been used in a range of issues relevant to nursing and healthcare, for example, theory in qualitative research [23] and vicarious trauma in child protection research [24].

A FMRS seeks to identify what is happening within a particular subject or field of inquiry; hence the search is restricted to a particular time period and to pre-identified journals. The review has four distinct features: It: 1) focuses on identifying trends in an area rather than a body of evidence; 2) creates a descriptive map or topography of key features of research within the field rather than a synthesis of findings; 3) comments on the overall approach to knowledge production rather than the state of the evidence; 4) examines this within a broader epistemological context. These are translated into three specific focused activities: 1) targeted journals; 2) a specific subject; 3) a defined time period. The FMRS therefore, is distinct from other forms of review because it responds to questions concerned with ‘what is happening in this field?’ It was thus an ideal method to investigate current practices in literature reviews in nursing.

Using the international Scopus (2016) SCImago Journal and Country Rank, we identified the five highest ranked journals in nursing at that time of undertaking the review. There was no defined method for determining the number of journals to include in a review; the aim was to identify a sample and we identified five journals in order to search from a range of high ranking journals. We discuss the limitations of this later. Journals had to have ‘nursing’ or ‘nurse’ in the title and we did not include journals with a specialist focus, such as nutrition, cancer etcetera. The included journals are shown in Table 2 and are in order according to their ranking. We recognise that our journal choice meant that only articles published in English made it into the review.

**Table 2:** Included journals

International Journal of Nursing Studies (IJNS) (UK).	
Nurse Education Today (UK).	
Nursing Ethics (USA).	
Journal of Advanced Nursing (UK).	
Journal of Nursing Management (UK).	

A key decision in a FMRS is the time-period within which to retrieve relevant articles. Like many other forms of review, we undertook an initial scoping to determine the feasibility and parameters of the project [19]. In our previous reviews, the timeframe has varied from three months [23] to 6 years [24]. The main criterion is the likelihood for the timespan to contain sufficient articles to answer the review questions. We set the time parameter from January 2017–June 2018. We each took responsibility for two and three journals each from which to retrieve articles. We reviewed the content page of each issue of each journal. For our purposes, in order to reflect the diverse range of terms for describing a literature review, as described earlier in this paper, any paper that contained the term ‘review’ or ‘synthesis’ in the title was included in the review. This was done by each author individually but to enhance rigour, we worked in pairs to check each other’s retrieval processes to confirm inter-rater consistency. This process allowed any areas of uncertainty to be discussed and agreed and we found this form of calibration crucial to the process. The inclusion and exclusion criteria are shown in Table 3.

**Table 3:** Inclusion and exclusion criteria for papers included in the focused mapping review and synthesis

Include	Exclude
Word ‘review’ or ‘synthesis’ in the title	Policy and book reviews
Report on a form of review of literature	Concept analyses

Articles meeting the inclusion criteria, papers were read in full and data was extracted and recorded as per the proforma developed for the study (Table 4). The proforma was piloted on two papers to check for usability prior to data extraction. Data extraction was done independently but we discussed a selection of papers to enhance rigour of the process. No computer software was used in the analysis of the data. We did not critically appraise the included studies for quality because our purpose was to profile what is happening in the field rather than to draw conclusions from the included studies’ findings.

**Table 4:** Data extraction table

Journal reference:	What type of review is recorded in the title of the paper?	Was the search strategy comprehensive? (for example, does the methods section record multiple databases searches and additional strategies such as scrutiny of reference lists?)	What types of papers were included in the review? (for example, does the methods section record whether qualitative or quantitative papers were included?)	Was critical appraisal undertaken and used within the analysis? (for example, does the methods section record how critical appraisal has been used within the analysis or only that it has been undertaken?)	Was there a stated approach to data analysis? (for example does the methods section record a thematic or meta-analysis?).	Was there alignment between the stated type of review and the actual methods used within the study? (for example if an integrative review was stated, do the methods reflect this approach? [15]

Once the details from all the papers had been extracted onto the tables, we undertook an analysis to identify common themes in the included articles. Because our aim was to produce a snap-shot profile, our analysis was thematic and conceptual. Although we undertook some tabulation and numerical analysis, our primary focus was on capturing patterns and trends characterised by the proliferation era. In line with the FMRS method, in the findings section we have used illustrative examples from the included articles that reflect and demonstrate the point or claim being made. These serve as useful sources of information and reference for readers seeking concrete examples.

## Results

Between January 2017 and June 2018 in the five journals we surveyed, a total of 222 papers with either ‘review’ or ‘synthesis’ in the title were retrieved and included in our analysis. We identified three primary themes: 1) Proliferation in names for doing a review; 2) Allegiance to an established review method; 3) Clarity about review processes. The results section is organised around these themes.

### Proliferation in names for doing a review

We identified more than 35 terms used by authors to describe a literature review. Because we amalgamated terms such as ‘qualitative literature review’ and ‘qualitative review’ the exact number is actually slightly higher. It was clear from reading the reviews that many different terms were used to describe the same processes. For example qualitative systematic review, qualitative review and meta-synthesis, qualitative meta-synthesis, meta-ethnography all refer to a systematic review of qualitative studies. We have therefore grouped together the review types that refer to a particular type of review as described by the authors of the publications used in this study (Table 5).

**Table 5:** An overview of review types as indicated in the title of papers included in this study

1. Systematic reviews of quantitative research (systematic literature review; systematic review; systematic review of quantitative research; systematic review and narrative synthesis): *n* = 82.	
2. Systematic reviews of qualitative and quantitative studies (integrative narrative review, mixed method review, systematic review of qualitative and quantitative evidence, mixed method meta-synthesis): *n* = 46.	
3. Systematic review +meta-analysis (Cochrane review summary): *n* = 29.	
4. Systematic review of qualitative studies (qualitative meta-synthesis; systematic review and meta-synthesis; systematic review of qualitative studies; meta-ethnography; critical interpretative synthesis): *n* = 27.	
5. Scoping review (including narrative scoping review): *n* = 15.	
6. Literature review (critical literature review; review; literature review, narrative review: *n* = 13.	
7. Realist review: *n* = 3.	
8. Rapid evidence/ rapid review of evidence: *n* = 2.	
9. Review of reviews (overview review; umbrella review): *n* = 2.	
10. Conceptual review: *n* = 1.	
11. Theoretical review: *n* = 1.	
12. Methodological review: *n* = 1.	

In many reviews, the specific type of review was indicated in the title as seen for example in Table 5. A striking feature was that all but two of the systematic reviews that contained a meta-analysis were labelled as such in the title; providing clarity and ease of retrieval. Where a literature review did not contain a meta-analysis, the title of the paper was typically referred to a ‘systematic review’; the implication being that a systematic review is not necessarily synonymous with a meta-analysis. However as discussed in the following section, this introduced some muddying of water, with different interpretations of what systematic review means and how broadly this term is applied. Some authors used the methodological type of included papers to describe their review. For example, a Cochrane-style systematic review was undertaken [25] but the reviewers did not undertake a meta-analysis and thus referred to their review as a ‘quantitative systematic review’.

### Alliegence to an established literature review method

Many literature reviews demonstrated alliegence to a defined method and this was clearly and consistently described by the authors. For example, one team of reviewers [26] articulately described the process of a ‘meta-ethnography’ and gave a detailed description of their study and reference to the origins of the method by Noblet and Hare (1988) [12]. Another popular method was the ‘integrative review’ where most authors referred to the work of one or two seminal papers where the method was originally described (for example, Whitemore & Knafl 2005 [15]).

For many authors the term systematic review was used to mean a review of quantitative research, but some authors [27–29],used the term systematic review to describe reviews containing both qualitative and quantitative data.

However in many reviews, commitment to a method for doing a literature review appeared superficial, undeveloped and at times muddled. For example, three reviews [30–32]^,^ indicate an integrative review in the title of their review, but this is the only reference to the method; there is no further reference to how the components of an integrative review are addressed within the paper. Other authors do not state allegiance to any particular method except to state a ‘literature review’ [33] but without an outline of a particular method for doing so. Anther review [34] reports a ‘narrative review’ but does not give further information about how this was done, possibly indicative of the lack of methods associated with the traditional narrative review. Three other reviewers documented how they searched, appraised and analysed their literature but do not reference an over-riding approach for their review [35–37]. In these examples, the review can be assumed to be a literature review, but the exact approach is not clear.

In other reviews, the methods for doing a literature review appear to be used interchangeably. For example in one review [38] the term narrative review was used in the title but in the main text an integrative review was described. In another review [39] two different and distinct methods were combined in a ‘meta-ethnographic meta-synthesis’.

Some authors [40, 41] referred to a method used to undertake their review, for example a systematic review, but did not reference the primary source from where the method originated. Instead a secondary source, such as a textbook is used to reference the approach taken [20, 42].

### Clarity about review processes

Under this theme we discerned two principal issues: searching and appraisal. The majority of literature reviews contain three components- searching, appraisal and analysis, details of which are usually reported in the methods section of the papers. However, this is not always the case and for example, one review [43] provides only a search strategy with no information about the overall method or how critical appraisal or analysis were undertaken. Despite the importance of the process of analysis, we found little discussion of this in the papers reviewed.

The overwhelming trend for those doing a literature review was to describe a comprehensive search; although for many in our sample, a comprehensive search appeared to be limited to a database search; authors did not describe additional search strategies that would enable them to find studies that might be missed through electronic searching. Furthermore, authors did not define what a comprehensive search entailed, for example whether this included grey literature. We identified a very small number of studies where authors had undertaken a purposive sample [26, 44]; in these reviews authors clearly stated that their search was for ‘seminal papers’ rather than *all* papers.

We reviewed the approaches to critical appraisal described in the papers and there were varying interpretations of what this means and which aspect of the included articles were to be subject to appraisal. Some authors [36, 45, 46] used the term ‘critical appraisal’ to refer to relevance of the paper to the review, rather than quality criteria. In that sense critical appraisal was used more as an inclusion criterion regarding relevance, rather than quality in the methods used. Mostly though, the term was used to describe the process of critical analysis of the methodological quality of included papers included in a review. When the term was used in this way to refer to quality criteria, appraisal tools were often used; for example, one review [47] provides a helpful example when they explain how a particular critical appraisal tool was used to asses the quality of papers in their review. Formal critical appraisal was undertaken by the vast majority reviewers, however the role of critical appraisal in the paper was often not explained [33, 48]. It was common for a lot of detail to be provided about the approach to appraisal, including how papers were assessed and how disagreements between reviewers about the quality of individual papers were resolved, with no further mention of the subsequent role of the appraisal in the review. The reason for doing the critical appraisal in the review was often unclear and furthermore, in many cases, researchers included all papers within their review regardless of quality. For example, one team of reviewers [49] explained how the process, in their view, is not to exclude studies but to highlight the quality of evidence available. Another team of reviewers described how they did not exclude studies on the basis of quality because of the limited amount of research available on the topic [50].

## Discussion

Our review has identified a multiplicity of similar terms and approaches used by authors when doing a literature review, that we suggests marks the ‘proliferation era’. The expansion of terms used to describe a literature review has been observed previously [19, 21]. We have identified an even wider range of terms, indicating that this trend may be increasing. This is likely to give the impression of an incoherent and potentially confusing approach to the scholarly undertaking of doing a literature review and is likely to be particularly problematic for novice researchers and students when attempting to grapple with the array of approaches available to them. The range of terms used in the title of papers to describe a literature review may cause both those new to research to wonder what the difference is between a qualitative evidence synthesis and a qualitative systematic review and which method is most suitable for their enquiry.

The clearest articles in our review were those that reported a systematic review with or without a meta-analysis. For example, one team of reviewers [25] undertook a Cochrane-style systematic review but did not undertake a meta-analysis and thus referred to their review as a ‘quantitative systematic review’. We found this form of labelling clear and helpful and is indeed in line with current recommendations [8]. While guidelines exist for the publication of systematic reviews [8, 51], given the range of terms that are used by authors, some may be unclear when these guidelines should apply and this adds some confusion to the field. Of course, authors are at liberty to call their review processes whatever they deem appropriate, but our analysis has unearthed some inconsistencies that are confusing to the field of literature reviewing.

There is current debate about the status of literature reviews that are not ‘Cochrane’ style reviews [52]. Classification can be complex and whilst it might be tempting to refer to all non Cochrane-style reviews as ‘narrative reviews’ [52], literature reviews that conform to a recognised method would generally not be considered as such [53] and indeed the Cochrane Collaboration handbook refers to the principles of systematic review as applicable to different types of evidence, not just randomised controlled trials [5] .This raises the question as to whether the term systematic review should be an umbrella term referring to any review with an explicit method; which is implicit in the definition of a systematic review, but which raises the question as to how rigorous a method has to be to meet these standards, a thorny issue which we have identified in this study.

This review has identified a lack of detail in the reporting of the methods used by those doing a review. In 2017, Thorne raised the rhetorical question: ‘What kind of monster have we created?‘ [54]. Critiquing the growing investment in qualitative metasyntheses, she observed that many reviews were being undertaken that position themselves as qualitative metasyntheses, yet are theoretically and methodologically superficial. Thorne called for greater clarity and sense of purpose as the ‘trend in synthesis research marches forward’ [54]. Our review covered many review types, not just the qualitative meta-synthesis and its derivatives. However, we concur with Thorne’s conclusion that research methods are not extensively covered or debated in many of the published papers which might explain the confusion of terms and mixing of methods.

Despite the proliferation in terms for doing a literature review, and corresponding associated different methods and a lack of consistency in their application, our review has identified how the methods used (or indeed the reporting of the methods) appear to be remarkably similar in most publications. This may be due to limitations in the word count available to authors. However for example, the vast majority of papers describe a comprehensive search, critical appraisal and analysis. The approach to searching is of particular note; whilst comprehensive searching is the cornerstone of the Cochrane approach, other aproaches advocate that a sample of literature is sufficient [15, 20]. Yet in our review we found only two examples where reviewers had used this approach, despite many other reviews claiming to be undertaking a meta-ethnography or meta-synthesis. This indicates that many of those doing a literature review have defaulted to the ‘comprehensive search’ irrespective of the approach to searching suggested in any particular method which is again indicative of confusion in the field.

Differences are reported in the approach to searching and critical appraisal and these appear not to be linked to different methods, but seem to be undertaken on the judgement and discretion of the reviewers without rationale or justification within the published paper. It is not for us to question researchers’ decisions as regards managing the flow of articles through their reviews, but when it comes to the issue of both searching and lack of clarity about the role of critical appraisal there is evidence of inconsistency by those doing a literature review. This reflects current observations in the literature where the lack of clarity about the role of critical appraisal within a literature review is debated ^.^[55, 56].

Our review indicates that many researchers follow a very similar process, regardless of their chosen method and the real differences that do exist between published methods are not apparent in many of the published reviews. This concurs with previously mentioned concerns [54] about the superficial manner in which methods are explored within literature reviews. The overriding tendency is to undertake a comprehensive review, critical appraisal and analysis, following the formula prescribed by Cochrane, even if this is not required by the literature review method stated in the paper. Other researchers [52] have questioned whether the dominance of the Cochrane review should be questioned. We argue that emergence of different methods for doing a literature review in a systematic way has indeed challenged the perceived dominance of the Cochrane approach that characterised the dichotomy era, where the only alternative was a less rigourous and often poorly described process of dealing with literature. It is positive that there is widespread acknowledgement of the validity of other approaches. But we argue that the expansion era, whereby robust processes were put forward as alternatives that filled the gap left by polarisation, has gone too far. The magnitude in the number of different approaches identified in this review has led to a confused field. Thorne [54] refers to a ‘meta-madness’; with the proliferation of methods leading to the oversimplification of complex literature and ideas. We would extend this to describe a ‘meta-muddle’ in which, not only are the methods and results oversimplified, but the existence of so many terms used to describe a literature review, many of them used interchangeably, has added a confusion to the field and prevented the in-depth exploration and development of specific methods. Table 6 shows the issues associated with the proliferation era and importantly, it also highlights the recommendations that might lead to a more coherent reviewing community in nursing.

**Table 6:** Features of the proliferation era

The issue	Recommendations
Where articles are labelled as ‘systematic review’, interpretations vary. Because there are so many forms of review, this term might now be too broad and generic.	Make a specific statement about the type of review undertaken and provide explanation and critique of its use
Adherence to an established method is often poorly described and confused.	When choosing an established method, take time to understand it and follow its central tenets
Reliance on secondary sources, rather than reference to original texts, leading to misunderstandings about some forms of review	Reference to original sources is important, particularly in higher-level academic reviews. The reading and citation of subsequent texts should provide support and context, rather than the basis of understanding
Proliferation of terms to describe approaches (particularly a feature of qualitative reviews)	Consolidation is required, with limitation of review types named
Many researchers appear to undertake the same processes, irrespective of what they call the review	Greater understanding of types of reviews is necessary and higher levels of explanation and justification of the processes undertaken
Comprehensive searchers are undertaken when the stated review type does not suggest this is necessary	Not all reviews require comprehensive searches but they appear to be the mainstream. Greater confidence in not using such searches is required
Critical appraisal is understood to mean different things and the purposes are unclear	Better levels of understanding and explanation of the purposes and outcomes of critical appraisal are required

The terms used for doing a literature review are often used both interchangeably and inconsistently, with minimal description of the methods undertaken. It is not surprising therefore that some journal editors do not index these consistently within the journal. For example, in one edition of one journal included in the review, there are two published integrative reviews. One is indexed in the section entitled as a ‘systematic review’, while the other is indexed in a separate section entitled ‘literature review’. In another edition of a journal, two systematic reviews with meta-analysis are published. One is listed as a research article and the other as a review and discussion paper. It seems to us then, that editors and publishers might sometimes also be confused and bewildered themselves.

Whilst guidance does exist for the publication of some types of systematic reviews in academic journals; for example the PRISMA statement [8] and Entreq guidelines [51], which are specific to particular qualitative synthesis, guidelines do not exist for each approach. As a result, for those doing an alternative approach to their literature review, for example an integrative review [15], there is only general publication guidance to assist. In the current reviewing environment, there are so many terms, that more specific guidance would be impractical anyway. However, greater clarity about the methods used and halting the introduction of different terms to mean the same thing will be helpful.

### Limitations

This study provides a snapshot of the way in which literature reviews have been described within a short publication timeframe. We were limited for practical reasons to a small section of high impact journals. Including a wider range of journals would have enhanced the transferability of the findings. Our discussion is, of course, limited to the review types that were published in the timeframe, in the identified journals and which had the term ‘review’ or ‘synthesis’ in the title. This would have excluded papers that were entitled ‘meta-analysis’. However as we were interested in the range of reviews that fall outside the scope of a meta-analysis, we did not consider that this limited the scope of the paper. Our review is further limited by the lack of detail of the methods undertaken provided in many of the papers reviewed which, although providing evidence for our arguments, also meant that we had to assume meaning that was unclear from the text provided.

## Conclusion

The development of rigorous methods for doing a literature review is to be welcomed; not all review questions can be answered by Cochrane style reviews and robust methods are needed to answer review questions of all types. Therefore whilst we welcome the expansion in methods for doing a literature review, the proliferation in the number of named approaches should be, in our view, a cause for reflection. The increase in methods could be indicative of an emerging variation in possible approaches; alternatively, the increase could be due to a lack of conceptual clarity where, on closer inspection, the methods do not differ greatly and could indeed be merged. Further scrutiny of the methods described within many papers support the latter situation but we would welcome further discussion about this. Meanwhile, we urge researchers to make careful consideration of the method they adopt for doing a literature review, to justify this approach carefully and to adhere closely to its method. Having witnessed an era of dichotomy, expansion and proliferation of methods for doing a literature review, we now seek a new era of consolidation.
